# Mir363-3p Treatment Attenuates Long-Term Cognitive Deficits Precipitated by an Ischemic Stroke in Middle-Aged Female Rats

**DOI:** 10.3389/fnagi.2020.586362

**Published:** 2020-09-29

**Authors:** Aditya Panta, Karienn Montgomery, Marissa Nicolas, Kathiresh K. Mani, Dayalan Sampath, Farida Sohrabji

**Affiliations:** Women’s Health in Neuroscience Program, Department of Neuroscience and Experimental Therapeutics, Texas A&M Health Science Center College of Medicine, Texas A&M University, Bryan, TX, United States

**Keywords:** microRNA, cognitive impairment, Barnes maze, novel object recognition test, social interaction, myelin, gait

## Abstract

Cognitive impairment and memory loss are commonly seen after stroke and a third of patients will develop signs of dementia a year after stroke. Despite a large number of studies on the beneficial effects of neuroprotectants, few studies have examined the effects of these compounds/interventions on long-term cognitive impairment. Our previous work showed that the microRNA mir363-3p reduced infarct volume and sensory-motor impairment in the acute stage of stroke in middle-aged females but not males. Thus, the present study determined the impact of mir363-3p treatment on stroke-induced cognitive impairment in middle-aged females. Sprague–Dawley female rats (12 months of age) were subjected to middle cerebral artery occlusion (MCAo; or sham surgery) and injected (iv) with mir363-3p mimic (MCAo + mir363-3p) or scrambled oligos (MCAo + scrambled) 4 h later. Sensory-motor performance was assessed in the acute phase (2–5 days after stroke), while all other behaviors were tested 6 months after MCAo (18 months of age). Cognitive function was assessed by the novel object recognition test (declarative memory) and the Barnes maze (spatial memory). The MCAo + scrambled group showed reduced preference for a novel object after the stroke and poor learning in the spatial memory task. In contrast, mir363-3p treated animals were similar to either their baseline performance or to the sham group. Histological analysis showed significant deterioration of specific white matter tracts due to stroke, which was attenuated in mir363-3p treated animals. The present data builds on our previous finding to show that a neuroprotectant can abrogate the long-term effects of stroke.

## Introduction

Cognitive impairment is a common consequence after a stroke that significantly impairs the patient’s functional recovery as well as the quality of life. More than half of stroke patients suffer from some level of cognitive impairment by 6 months after stroke (Jacquin et al., 2014; Mellon et al., 2015). Patients who do not develop cognitive impairment directly after stroke have a 9-fold increased risk of developing delayed cognitive impairment (Kokmen et al., 1996). Age is an important risk factor for both stroke as well as cognitive decline and studies have shown that prevalence of cognitive decline after stroke increases exponentially with age (Gorelick et al., 2011). In addition to age, previous or recurrent stroke, the volume of infarction, location of the stroke, and female sex are all strongly associated with post-stroke cognitive impairment (Pendlebury and Rothwell, 2009). In fact, among these survivors, women are more likely to require assisted living facilities because of deteriorating cognitive function (Gall et al., 2012; Bushnell and McCullough, 2014; Bushnell et al., 2014).

The precise mechanisms underlying post-stroke cognitive dysfunction are still not well known, but studies have suggested that cognitive dysfunction could be due to an ischemic insult to the brain parenchyma, vasculature as well as the white matter. The prefrontal cortex is an important region for cognitive functioning, providing an executive, “top-down” control on cognition (Shimamura et al., 1995; Sakai et al., 2002; Postle, 2006). Accordingly, ischemic injury to the pre-frontal cortex induces cognitive dysfunction (Livingston-Thomas et al., 2015). The medial entorhinal cortex (MEC) provides input to the hippocampus for spatial information, whereas the lateral entorhinal cortex (LEC) provides input for nonspatial (contextual) information (Hargreaves et al., 2005; Wilson et al., 2013). However, these remote regions are vulnerable not only to focal ischemia but also to distal ischemia. Middle cerebral artery occlusion (MCAo), which comprises 75% of all strokes, primarily affects the striatum and the overlying cortex, but can also affect remote brain regions in a progressive manner. Delayed neuronal death (DND) in the CA1 region of the hippocampus, reduced activity in the prefrontal cortex, and thinning of the entorhinal cortex have all been reported after middle cerebral artery ischemia (Kirino, 1982; States et al., 1996; Butler et al., 2002; Wang et al., 2004; Paradiso et al., 2011). Li and colleagues have shown that MCAo leads to increased GABAergic neurotransmission and reduced activity of extracellular regulated protein kinase (ERK) in both hippocampi resulting in cognitive deficits. Similarly, an increased inflammatory response, in the form of activated microglia, is observed in the hippocampus of rats that have cognitive impairment after MCAo (Ward et al., 2018). These results were supported by the findings of Gemmell and colleagues where hippocampal atrophy was observed in the post mortem brains of patients with delayed post-stroke dementia (Gemmell et al., 2012). Collectively, DND in brain regions that are remote from the primary occlusion may explain the delayed onset of cognitive impairment in stroke patients.

Our previous studies showed that in the chronic phase (1–3 months later) after MCAo, middle-aged female rats exhibited depressive-like symptoms in the form of anhedonia, increased “despair,” and decreased social interaction (Panta et al., 2019). This was accompanied by transiently elevated levels of inflammatory cytokines and lower circulating levels of Brain-derived neurotrophic factor, and attrition of the projection pathway between midbrain neurons of the ventral tegmental area/substantia nigra to the striatum (Panta et al., 2019). However, non-spatial memory as assessed by the Novel Object Recognition Task (NORT) was not impaired after MCAo at 3 months. Given the delayed onset of cognitive impairment reported for stroke patients, the present study tested the hypothesis that MCAo would affect cognitive function much later in the chronic phase of stroke and that early treatment with mir363-3p would attenuate these cognitive impairments.

The choice of mir363-3p as an intervention is based on our three previous studies. A plasma miRNA profiling study of adult and middle-aged animals revealed that mir363-3p was significantly inversely correlated with infarct volume (Selvamani et al., 2014; Selvamani and Sohrabji, 2017). To directly assess if mir363-3p was mechanistically linked to stroke outcomes, middle-aged females were injected with an antagomir to mir363-3p 4 h after the onset of ischemia. We reported that mir363-3p, injected iv, is readily taken up neurons in the ischemic hemisphere and that this treatment significantly decreased infarct volume and reduced motor impairment (Selvamani and Sohrabji, 2017). MiR363 is identified as a tumor suppressor in various cancers (Floyd et al., 2014; Song et al., 2015; Wang et al., 2016) and is predicted to repress caspase-3, the cell death effector (Floyd et al., 2014). In our stroke studies, mir363-3p mimics reduced both caspase-3 expression and activity in the ischemic brain (Selvamani and Sohrabji, 2017). Subsequently, we reported that mir363-3p treatment significantly reduced the depressive phenotype that develops in the chronic phase (30–90 days) after stroke (Panta et al., 2019). In this study, we show that mir363-3p treatment also improves long term cognitive impairment induced by stroke.

## Materials and Methods

### Ethics Statement

All experimental protocols were approved by the Texas A&M University Institutional Animal Care and Use Committee. All animal use was conducted following the Guide for the Care and Use of Laboratory Animals and PHS Policy on Humane Care and Use of Laboratory Animals. The TAMU animal care program is AAALAC accredited. All experimental procedures and reporting of data follow the ARRIVE guidelines (Kilkenny et al., 2010).

### Animals

This study utilized middle-aged female rats, based on our prior work that mir363-3p is neuroprotective for females but not males (Selvamani and Sohrabji, 2017). Middle-aged (12 months old, 260–320 g) were purchased from Envigo (IN) and were housed in an AAALAC-accredited vivarium on a 12/12 light/dark cycle with controlled temperature (22°C) and humidity (45–55%). Food and water were available *ad libitum*. The rats were allowed to acclimatize to the vivarium for 3 weeks after arrival and daily vaginal swabs were performed up to 21 days to determine the estrus cycle as described in our previous work (Jezierski and Sohrabji, 2001; Selvamani and Sohrabji, 2010). When the animals displayed cell cytology consistent with the diestrus phase for at least seven consecutive days, they were included in the study. Our previous works have shown that female rats in constant diestrus have low estradiol and elevated FSH levels (Jezierski and Sohrabji, 2001; Selvamani and Sohrabji, 2010), a hormonal pattern that resembles the hormonal profile of post-menopausal women. These animals are referred to as middle-aged due to the natural life span of the Sprague Dawley female (Davis et al., 1956) although their key characteristic for inclusion is reproductive senescence (acyclicity). A total of 30 animals were used. Animals were randomly assigned to sham (*n* = 10) or MCAo surgery (*n* = 20). After MCAo surgery, animals were randomly assigned to one of two treatment groups (scrambled, *n* = 10; or mir363-3p *n* = 10). All the behavioral tests were performed between 8:00 am and 12:00 pm and experimenters were blinded to the treatment condition while performing and analyzing the tests.

### Middle Cerebral Artery Occlusion (MCAo)

Animals were subjected to MCAo by stereotaxic injection of a vasoconstrictor [Endothelin-1 (ET-1), using our established protocol (Balden et al., 2012; Selvamani et al., 2012, 2014)]. The ET1 model causes a gradual constriction of the vessel which lasts approximately 16 h in the cortex and 7 h in the striatum (Biernaskie et al., 2001; Selvamani and Sohrabji, 2010). Slow ET1-mediated constriction and relaxation of MCA mimic the natural history of some types of ischemic strokes in human populations which transition from hypo-perfusion to ischemia followed by slow reperfusion with clot dissolution. Animals were anesthetized with a mixture of ketamine (87 mg/kg) and xylazine (13 mg/kg) and placed in stereotaxic equipment. Endothelin-1 (American Peptide Company; 0.5 μg/μl, 600 pmol; 3 μl) was microinjected at the following coordinates relative to Bregma: Anterior-posterior (AP): +0.9, Medio-lateral (ML): −3.4, Dorso-ventral (DV): −8.5 to occlude left middle cerebral artery. Throughout surgery, body temperature was maintained at 37°C. For sham surgeries, animals were subject to all the surgical procedures (anesthetic, scalp incision, drilling of the skull), but did not receive ET-1. All animals were single-housed after MCAo.

#### miRNA Treatment

Four hours after MCAo, animals received either scrambled oligos or mir363-3p (7 mg/kg, 300 μl) administered *via* tail vein injection. Scrambled and mir363-3p mimic (AAUUGCACGGUAUCCAUCUGU) oligonucleotides were purchased from Thermo Fisher, Grand Island, NY, and packaged in *in vivo* RNA-LANCEr II kit (Bio-Scientific, Austin, TX, USA).

### Behavioral Assays

All behavior tests were performed between 8:00 am and 12:00 pm in the light cycle. For assessing sensorimotor function, an adhesive tape removal test was performed before (Pre, −2 days) and after (2 and 5 days) stroke. Assays for non-spatial memory and social interaction were performed before (−10 days) and after (6 months) stroke. Spatial memory was tested only after (6 months) stroke. Gait was assessed at the end of the study using DigiGait equipment. All tests were performed and scored by experimenters who were blind to the treatment condition.

#### Adhesive-Tape Removal Test

This test was performed as described in our previous works (Selvamani et al., 2012, 2014) and is a robust and reliable test of stroke-induced sensory-motor impairment in the acute phase of the stroke. A square adhesive tape (12.7 × 12.7 mm) was attached on the palmar side of the paw of each forelimb. The time taken to remove the tape was recorded for three trials. Each trial had a maximum time limit of 120 s and the animals were allowed to rest for at least 5 min between the trials.

#### Social Interaction Test

This test was performed as described in our previous study (Panta et al., 2019). Briefly, a three-chambered Plexiglass box was used where each chamber had a dimension of 40″ × 13″ and all chambers had open access to each other. For habituation, the three chambers were closed off from each other, and the test rat was placed in each chamber for 2 min. Thereafter, the rat was allowed to freely explore the three chambers for an additional 10 min and then returned to the home cage. For the testing session, a conspecific (stranger) rat was placed within a plastic mesh cylinder in one of the end chambers, while the test rat was placed back in the middle chamber and allowed to explore for 10 min. The time spent in each chamber was recorded and sociability was scored as the total time (in seconds) spent by the test rat in the chamber with the stranger rat.

#### Novel Object Recognition Test (NORT)

This test was performed as described in our previous study (Panta et al., 2019). This test consisted of three phases: habituation, familiarization, and test phase. In the habituation phase, the rat was placed in a 16″ × 16″ open field and allowed to freely explore the arena for 10 min each day for 2 days as described in (Panta et al., 2019). On the third day (familiarization phase), the animal was again placed in the open-field apparatus, which now contained two identical objects (A + A) placed diagonally from each other. The rat was allowed to explore the arena and the objects for 10 min. The rat was then returned to its home cage for 1 h (retention interval) and then placed again in the open-field arena for the test phase. For the test phase, the arena contained two objects in the same location, one that was previously available (A) and the other that was novel (B). The rat’s behavior was recorded for 5 min and the amount of time spent exploring the novel object was determined from these recordings by an investigator blind to the experimental condition. Exploration of an object was defined as the rat sniffing or touching the object with its snout at a distance of <2 cm from the object. Climbing or sitting on the object was not defined as exploration. The discrimination index (DI) was calculated as follows (Antunes and Biala, 2012) DI: [*time spent with novel object*] − [*time spent with familiar object*]/[*time spent with novel object*] + [*time spent with familiar object*].

DI scores can range from −1 to +1. Animals with a negative DI at baseline (pre) were not included in further analysis of this test. Accordingly, one animal each group was excluded based on this criterion. Group differences were analyzed by the Kruskal Wallis test comparing pre and post-stroke DI.

#### Barnes Maze

This test was performed to assess spatial memory (Rosenfeld and Ferguson, 2014). A circular maze (diameter of 48″) consisting of 20 holes was used. Each hole consisted of either a small square box (19 total; 4″ × 4″ × 2″) or one bigger escape box (8″ × 4″ × 4″). The test was divided into two phases spanning 4 days: habituation (1 day) and learning (3 days). For habituation, rats were placed in an escape box for 2 min covered with a lid. After 2 min in the escape box, rats were placed inside a dark tube at the center of the Barnes maze. Bright lights (for aversion) were turned on and the center tube gently lifted off allowing the rats to freely explore the maze for a maximum of 5 min to find the escape box. If the rat did not find or enter the escape box in 5 min during the day of habituation, the experimenter manually guided the rat to the escape box. Once the rat entered the escape box, lights were turned off. The next day, during the learning phase, the escape box was placed at a fixed location under the escape hole (goal), and the rats were allowed a maximum of 2 min to find the escape. Each rat underwent three trials at an interval of 15 min. Ethovision software (Noldus) was used to analyze the latency to find the escape box as well as velocity and distance traveled during each trial. Also, search strategies used by each group on each day were analyzed. Search strategies were binned into three broad categories: hippocampal-based (which includes “Direct,” “Corrected,” “Focused,” and “Long Correction”), non-hippocampal based (Serial) or unsuccessful strategies which include Random or Fail (Harrison et al., 2006; Rosenfeld and Ferguson, 2014; Illouz et al., 2016).

#### Digigait

Gait dynamics were recorded using ventral plane videography, as described in other works (Hampton et al., 2004; Kale et al., 2004). Briefly, on the first testing day, rats were exposed to the treadmill compartment (7 × 30 cm, DigiGait Imaging System, Mouse Specifics, Inc., Boston, MA, USA) to acclimate. The following day, animals were allowed to explore the compartment for 1 min with the motor speed set at 20 cm/s. Video images of the rat were collected at ~125 frames per second by a high-speed digital video camera mounted below the transparent treadmill belt. Most animals walked comfortably at this speed and it was sufficiently fast to prevent the rats from rearing or turning around during videography. Digigait indices such as brake, propel, and paw angle was analyzed as described by Piesla et al. (2009) using proprietary software.

### Histology

#### Tissue Processing

On completion of the behavioral experiments, rats were terminated humanely by anesthetic overdose and perfused transcardially with saline followed by 4% paraformaldehyde. The brains were removed from the skull and stored in a sucrose (10%) and sodium azide (0.01%) solution for a week before further processing. The brains were transferred to NSA Associates (TN, USA) for block embedding, sectioning, and staining. Brains were sectioned at 40 microns through the rostrocaudal extent of the brain. Every 10th section was processed for the Weil myelin stain to visualize myelinated axons.

#### Analysis of Myelinated Tracts

The main white matter tracts targeted for quantitation were the corpus callosum, the internal capsule, anterior commissure, and the lateral olfactory tract. Sections were first scanned for the rostrocaudal extent of each white matter regions of interest (ROI’s), based on standardized stereotactic coordinates of the rat brain atlas (Paxinos and Watson, 2004), referenced from Bregma: corpus callosum AP = +3.00 mm to AP = −1.72 mm; internal capsule: AP = 0.00 mm to AP = −4.20 mm); lateral olfactory tract: AP = 3.00 mm to AP = −01.32 mm, and anterior commissure: AP = 3.00 mm to AP = 00.00 mm. Sections were imaged at 2.5× magnification (Leica DM 6B) and processed in the ImageJ2 software (NIH), and the plugins provided in the FIJI’s (for ImageJ2) software package to enhance their contrast in a standardized manner. The high contrast images were then binarized into 8-bit images to segment the background, gray matter, and white matter separately. The myelinated corpus callosum, internal capsule, and other tracts were then clearly demarcated, and its outlines marked for quantification for each region of interest (ROI), on both the left (ischemic) hemisphere and the right (non-ischemic) hemisphere. An area measurement was taken at every 10th section (400 microns apart) and summed to derive the volume of myelin-positive staining. The volume of each tract on the ischemic hemisphere was normalized to the non-ischemic hemisphere.

#### Statistics

Data was tested for normality using the Shapiro–Wilk test and a *p*-value greater than 0.05 was considered to have passed the normality test. For all assays, group mean ± SD are reported. Group differences were determined by a one-way ANOVA (Sham, MCAo + scrambled, MCAo + mir363-3p), with planned comparisons (myelin analysis). For analyses where two time-points were compared (Social interaction, Barnes maze), a two-way ANOVA repeated measures design was used followed by planned comparisons pre and post-stroke. All group differences were considered significant at *p* < 0.05. Data were analyzed using Prism GraphPad (GraphPad, San Diego, CA, USA). Sample size: Each group initially had 10 animals; of the group that received MCAo, seven survived in the MCAo + scrambled group, while 10 survived in the MCAo + mir363-3p group. Seven of the 10 shams survived to the 18–20 months of age testing period. Due to the multiple tests and the advanced age of these animals, an occasional animal did not complete all parts of the test. Thus, in most behavioral assays, sample sizes range from 6 to 9. Animals were terminated at 22 months for histological analysis, where due to further age-related attrition, the sample size was sham = 4, MCAo + scrambled = 4, and MCAo + mir363-3p = 7.

## Results

In previous studies (Selvamani and Sohrabji, 2017; Panta et al., 2019), we found no differences in sham animals that received either the scrambled control or mir363-3p, on either sensory-motor tests, tests of depression, or NORT. Thus, for this study, the sham-scrambled and sham-mir363-3p groups were reduced and reported as the combined “Sham” group. Since the effects of mir363-3p treatment on infarct volume are already published (Selvamani and Sohrabji, 2017), they were not duplicated here. Furthermore, we have reported that mir363-3p mimic can be detected in circulation for 48 h and in the brain for up to 5 days, and then return to basal levels. Mir363-3p also improved sensory-motor impairment in two previous studies (Selvamani and Sohrabji, 2017; Panta et al., 2019) and this was confirmed in the present study before the long-term behavioral analysis. Sensorimotor deficits were assessed by the adhesive tape removal test, where latency to tape removal from the paw contralateral is increased after MCAo, and this measure is well correlated with the extent of infarction. As shown in Supplementary Figure 1, animals removed the tape rapidly before stroke but were significantly delayed in the early acute phase, i.e., 2 days and 5 days (*F*_(2,30)_= 47.13, *p* < 0.0001) with a treatment effect (*F*_(1,15)_ = 4.95, *p* = 0.0419). *Post hoc* analysis shows significant recovery in the MCAo + mir363-3p group as compared to MCAo + scrambled at day 5 (*p* = 0.0411). This data was consistent with our two previous studies that showed mir363-3p acutely improves sensorimotor deficits (Selvamani and Sohrabji, 2017; Panta et al., 2019).

All other tests described below were performed 6 months after MCAo.

### Social Interaction

**Figure 1:**
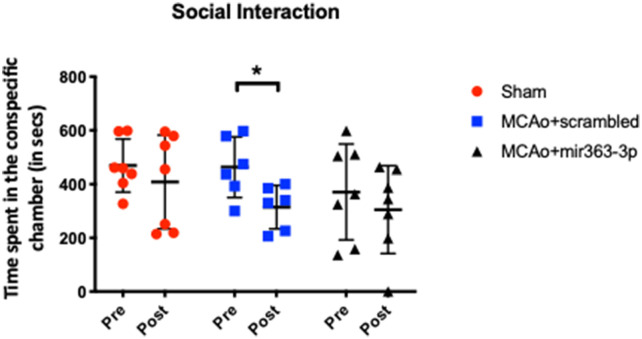
Effect of middle cerebral artery occlusion (MCAo) and microRNA treatment on social interaction. Sham animals and stroke animals treated with either scrambled oligos (MCAo + scrambled) or mir363-3p (MCAo + mir363-3p) were tested for social interaction with a stranger rat in a three-chamber apparatus before (Pre) and 6 months after (Post) MCAo. The histogram depicts the mean + SD of interaction time (in seconds). Key: *p < 0.05, comparison of the group with its baseline. *N* = 6–7/group.

Interaction with a conspecific was obtained for all three groups before (pre) and at 6 months after (post) stroke (Figure 1). Reduced interaction with a conspecific is indicative of social disinterest and loss of social cognition. Prior to stroke (or sham surgery), interaction with the conspecific was similar in the sham (469 + 98.5 s), MCAo + scrambled (463 + 112 s) and MCAo + mir363-3p (370 + 178 s) groups. Six months later, social interaction for the sham group decreased slightly (13%), which was not statistically different from prior interaction levels (paired *t*-test, *p* = 0.543). In contrast, the MCAo + scrambled group showed a 32% decline in interaction, which was significantly lower than pre-stroke levels (paired *t*-test, *p* = 0.035). However, the MCAo + mir363 showed a statistically insignificant decline (16%, *p* = 0.412) from baseline levels at 6 months after stroke. This is consistent with our earlier work showing social interaction declines after stroke (3 months) and is attenuated in the group that received mir363-3p.

### Cognitive Deficit After Stroke and Improvement by mir363-3p

#### Novel Object Recognition Test

**Figure 2:**
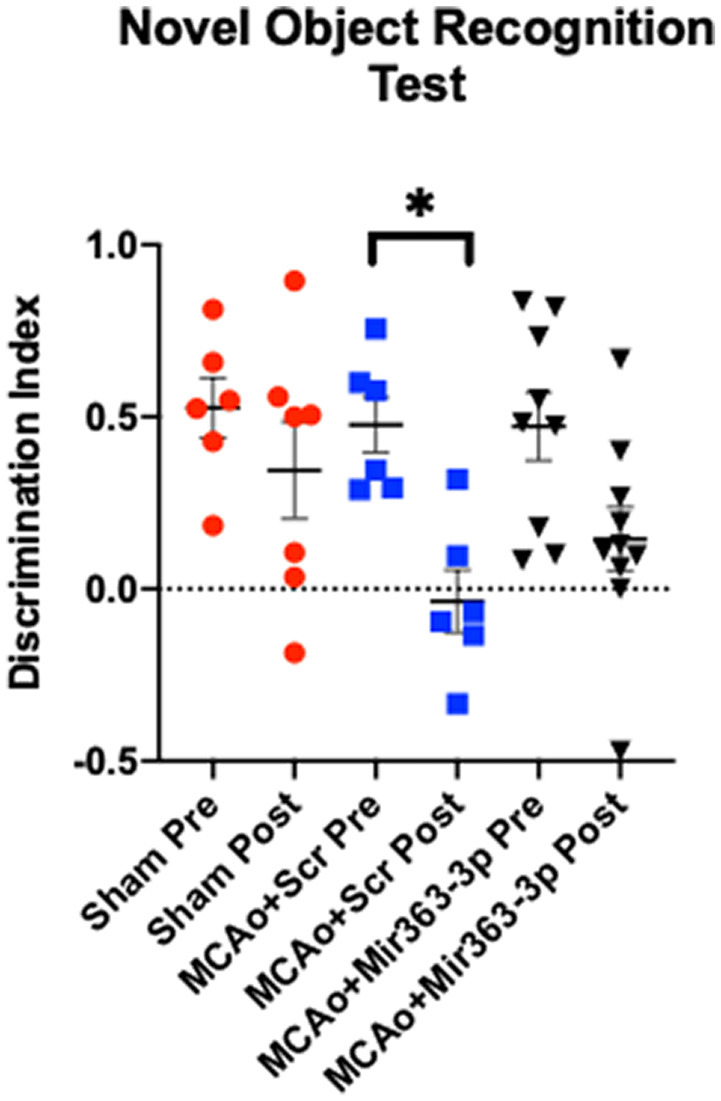
Effect of MCAo and microRNA treatment on the Novel Object Recognition Test. Sham animals and stroke animals treated with either scrambled oligos (MCAo + Scrambled) or mir363-3p (MCAo + mir363-3p) were tested on their preference for novel objects before (Pre) and 6 months after (Post) MCAo. The histogram depicts the mean Discrimination Index (DI) + SD preference for the novel object. Key: *p < 0.05, *N* = 6–9.

Preference for a novel object over a familiar object, indicated by the amount of time spent exploring the object, was assessed before (pre) and 6 months after MCAo/sham (post) surgery (Figure 2). Increased time spent exploring the novel object indicates retention of the memory of the familiar object and thus the ability to discriminate between the two objects. Preference was determined by the discrimination index, where a positive number indicates a preference for the novel object. Prior to stroke, all groups showed a similar level of preference for the novel object (sham: +0.52 ± 0.21, MCAo + scrambled: +0.47 ± 0.19, MCAo + mir363-3p: +0.47 ± 0.29). Six months after stroke, there was no difference in the DI of the Sham group (*p* = 0.8456). However, discrimination of the novel object was significantly reduced in the MCAo + scrambled group (*p* = 0.0129), indicating that stroke affects this non-spatial learning task. In contrast, the DI at 6 months after stroke in the group that received mir363-3p was no different from the baseline (pre-stroke) level (*p* = 0.1367). This data shows that early treatment with a neuroprotectant preserves long-term non-spatial cognitive loss in animals after stroke.

#### Barnes Maze

**Figure 3:**
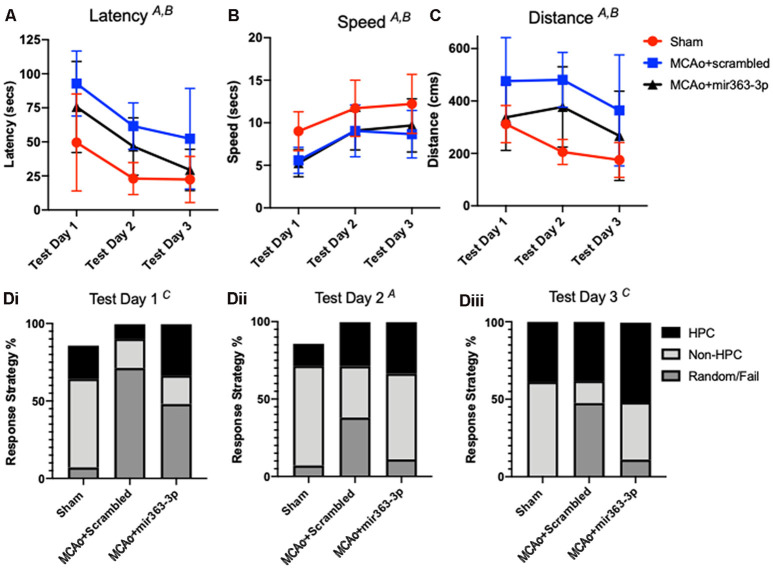
Effect of MCAo and microRNA treatment on the Barnes Maze Test. Sham animals and stroke animals treated with either scrambled oligos (MCAo + scrambled) or mir363-3p (MCAo + mir363-3p) were tested for **(A)** latency, **(B)** distance, and **(C)** speed in the Barnes maze 6 months after stroke. **(D)** The strategy used by each group on the 3 days of test **(i–iii)**. Data is presented as the mean + SD. Key: **(A)** main effect of time; **(B)** main effect of treatment; and **(C)** interaction effect, *N* = 7.

Spatial learning in the animals was assessed by latency to find an escape hole in a circular maze over 3 days (Figure 3A). Decreased latency over the testing days is indicative of learning, while longer latencies indicate impaired ability to locate the escape hole. There was a main effect of time (*F*_(2,40)_ = 22.13, *p* < 0.001), as well as a main effect of treatment on latency (*F*_(2,20)_ = 6.72, *p* = 0.0059). Planned comparisons showed that both MCAo groups initially had a higher latency, as compared to shams. On day 3, the MCAo + scrambled group took significantly longer than sham animals to find the escape hole (*p* = 0.0141), while latency for the MCAo + mir363-3p was not different from sham (*p* = 0.7894). To ensure that differences in latency were not due to impaired physical ability, velocity measurements were also analyzed for each trial day. As shown in Figure 3B, all groups were faster at finding (speed) the escape hole over time (the main effect of time, *F*_(2,40)_ : 22.6, *p* < 0.0001), however the sham animals were faster than either of the MCAo groups (scrambled or mir363-3p; *F*_(2,20)_ = 5.2, *p* = 0.0152). Distance traveled to reach the target (Figure 3C) was also affected by day (main effect of time, *F*_(2,40)_ : 5.365, *p* < 0.013) and by group (main effect of treatment, *F*_(2,20)_ : 7.944, *p* < 0.0029). Sham animals had the shortest distance traveled on each testing day, while the MCAo + scrambled group traversed the longest distance. Thus, while both MCAo groups are slower than the sham group, the MCAo + scrambled group has a greater travel distance indicating that cognitive impairment in this group is not due to motor deficits.

Group differences were also noted in the strategies used to find the escape hole. Strategies were binned over the three testing days for either hippocampal-based, non-hippocampal, or random/failure. On the first day (Figure 3Di), there was a group by strategy interaction effect (*F*_(4,60)_ : 5.681; *p* = 0.0006), such that both MCAo groups had greater random/failure events as compared to the sham group (Figure 3Di). On Day 2 (Figure 3Dii), group effects were not seen but strategy differences were noted (*F*_(2,40)_ : 4.747, *p* = 0.0141). Specifically, all groups showed more non-hippocampal based strategy than either hippocampal based or random/failure. On day 3 (Figure 3Diii), there was a group by strategy interaction effect (*F*_(4,60)_ : 3.505, *p* = 0.0123) such that MCAo + mir363-3p had similar, low random/fail strategy compared to sham (*p* = 0.7844), while the MCAo + scrambled group had significantly higher random/fail strategy as compared to sham (*p* = 0.0233). Overall, the strategy analysis complements the findings of the latency and distance data, in that the MCAo + scrambled group differs from sham, while MCAO + mir363-3p does not.

#### Gait Dynamics

**Figure 4:**
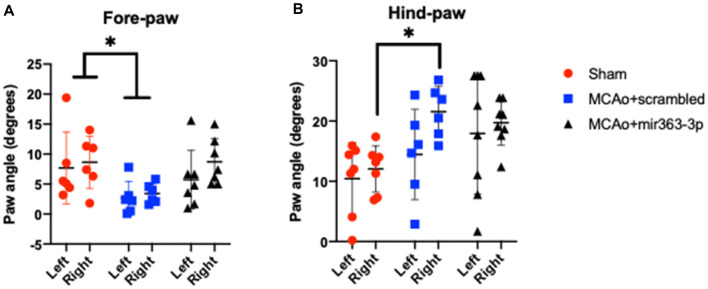
Effect of MCAo and microRNA treatment on gait dynamics. Sham animals and stroke animals treated with either scrambled oligos (MCAo + scrambled) or mir363-3p (MCAo + mir363-3p) were tested for several gait measures. Shown here are the paw angles (in degrees) for left and right forelimbs **(A)** and hindlimbs **(B)** 6 months after stroke. The histogram depicts mean + SD. Key: *p < 0.05, sham vs. MCAo + scrambled, *N* = 7–10.

Long term motor coordination after stroke was assessed by ventral plane videography (Digigait, Quincy, MA, USA) at the end of the study (Figure 4 and Supplementary Figure 2). Gait dynamics are crucial for regular behavior and are impaired in neurological diseases.

#### Paw Angle

Defined as the angle that the long axis of a paw makes with the direction of motion of the animal during peak stance. A narrower forelimb paw angle and wider hindlimb paw angle are associated with ataxia, spinal cord injury, and demyelinating diseases (Powell et al., 1999). There was an overall effect of treatment on the forelimb paw angle, such that the forelimb paw angle was significantly narrowed in the MCAo + scrambled group as compared to sham, on the ipsilesional and contralesional paw. No difference was seen between the MCAo + mir363-3p and sham (Figure 4A). For the hindlimb, the paw angle was not different across the groups on the ipsilesional paw, however contralesional paw angle was wider in the MCAo + scrambled group as compared to sham, while MCAo + mir363-3p treated animals were not different from sham (Figure 4B).

#### Brake Time

Defined as the duration between the initial paw contact and maximum paw contact while walking on the belt, longer brake times indicate precise control and distribution of the body load while walking. Brake time was no different among the groups (*p* = 0.6618) and across the limbs (Supplementary Figures 2A,B).

#### Propel

Defined as the duration of the maximum paw contact on the belt before swinging the limb again to take the next stride, shorter propel times indicate better strength and control over the body in motion. Propel duration was not different among the groups and across limbs (Supplementary Figures 2C,D).

#### Morphometry of Myelinated White Matter

**Figure 5:**
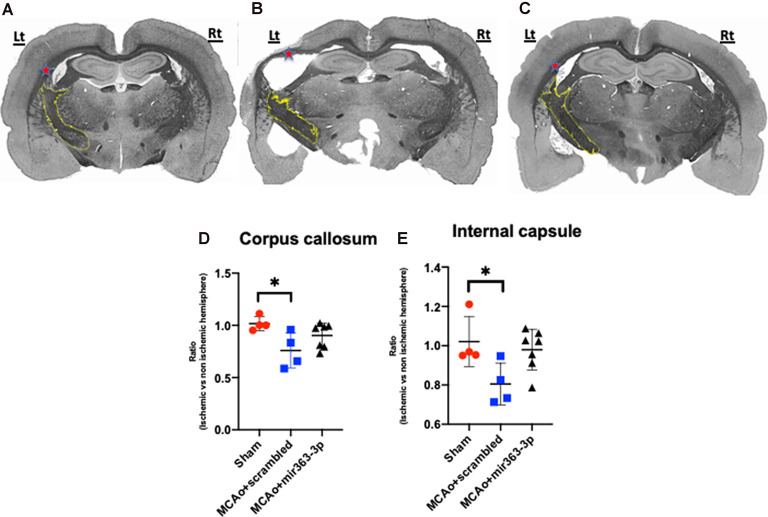
Effect of MCAo and microRNA treatment on myelin expression. Representative photomicrographs of Weil-stained coronal sections from sham **(A)**, MCAo + scrambled **(B)** and MCAo + mir363-3p groups **(C)**. Sections depict the corpus callosum (indicated by red *) and the internal capsule (outlined in yellow) on the ischemic (left) and non-ischemic (right) hemisphere. Histogram of normalized myelin ratio of the corpus callosum **(D)** and internal capsule **(E)** for each group. Bars represent mean ± SD. *N* = 4–7. *p < 0.05.

Four white matter tracts were analyzed and in each case, the volume of myelinated tissue on the ischemic hemisphere was normalized to the non-ischemic hemisphere. Shown in Figure 5 are representative sections of Weil-stained coronal sections from each group. In the sham group (Figure 5A), the corpus callosum (indicated by *) is bilaterally symmetrical, while in the MCAo + scrambled group (Figure 5B), the callosum is thinner and irregularly-shaped on the ischemic hemisphere as compared to the non-ischemic hemisphere. No asymmetry was seen in the MCAo + mir363-3p group (Figure 5C). This was confirmed by an overall effect of treatment on the myelin ratio (*F*_(2,11)_ = 5.124, *p* = 0.0268). Planned comparisons indicated a significant decrease in the myelin volume ratio in the MCAo + scrambled group as compared to sham (*p* = 0.0174; Figure 5D), while the ratio in the MCAo + mir363-3p group was no different from sham (*p* = 0.432) or the scrambled treated group (*p* = 0.19). A similar effect was seen in the normalized myelin ratio of the internal capsule (outlined in yellow; Figure 5A; main effect of treatment: *F*_(2,11)_ = 4.506, *p* = 0.0372). There was a significant decrease in the myelin ratio in the MCAo + scrambled group as compared to sham (*p* = 0.0458; Figure 5E), while the ratio of the MCAo + mir363-3p group was no different from sham (*p* = 0.703) or MCAo + scrambled group (0.071), although the latter indicated a trend. Thus, for these two white matter tracts, the mir363-3p group exhibited an intermediate state where the loss was not as notable as the scrambled treated group but did not return to baseline (sham) levels. There were no group differences in the inter-hemisphere myelin ratio of the anterior commissure or the lateral olfactory tract (Supplementary Figures 3A,B).

## Discussion

This study focused on long-term cognitive impairment after stroke in acyclic middle-age rats and the effect of microRNA treatment. The rationale for focusing on this age group is based on epidemiological observations that after menopause, women are at a higher risk of stroke as well as worse stroke outcomes as compared to young females and age-matched males (Reeves et al., 2008). Over one-third of these stroke survivors develop depression and some form of cognitive deficit over time, which significantly lowers their quality of life. Our previous work with mir363-3p shows that it is a stroke neuroprotectant for acyclic, middle-aged females but not age-matched males. Our main findings confirm that MCAo results in an early deficit in sensorimotor skills and long-term (6 months) impairment in cognition, including spatial and non-spatial memory. We also report that social interaction, which we previously reported is reduced at 3 months after stroke, is impaired at 6 months as well. Remarkably, early treatment with mir363-3p treatment abrogated cognitive deficits induced by stroke.

MicroRNAs have emerged as an attractive therapy for a wide range of diseases because of their role in regulating gene transcription and translation. These 18–25 nucleotide noncoding RNAs function by regulating the methylation of target genes (Lim et al., 2005), the stability of mRNA transcript (Denli and Hannon, 2003), or their translation to proteins (Ambros, 2001). MicroRNA profiles are altered by stroke as well as risk factors for stroke, as shown in clinical and preclinical studies (Dharap et al., 2011; Rink and Khanna, 2011; Jickling et al., 2014). Many preclinical studies have focused on microRNAs as a treatment for stroke, however, a vast majority of these studies used male animals exclusively (Sohrabji and Selvamani, 2019). We were among the first to use microRNA as a stroke neuroprotectant and have shown that neuroprotection from microRNAs has sex-specific effects. ICV administration of antagomirs to mir1 and let7f reduced infarct volume in female rats only, presumably by targeting the IGF-1 pathway (Selvamani et al., 2012). Similarly, intravenous administration of mir363-3p reduced infarct volume in middle-aged female rats but not males. Mir363-3p caused a sex-specific regulation of caspase-3, which has been shown previously as an effective target for stroke recovery in females but not males (Liu et al., 2011).

Few studies have focused on the role of microRNA in improving stroke outcomes beyond the acute phase. A clinical study in the Chinese cohort has shown that miR-132 expression in the blood is up-regulated in the cognitively-impaired stroke patients in both males and females (Huang et al., 2016). Liu et al. (2017) showed that miR134 improved cognition after stroke in males (age not specified). Similarly, inhibition of miR27b was shown to improve post-stroke cognition in 10 weeks old male mice (Wang et al., 2019). To the best of our knowledge, this present study is the first to address long-term cognitive deficits in a middle-aged female animal model, and to show that post-stroke microRNA treatment improves cognitive function long term after stroke. While the precise mechanism by which mir363-3p attenuates cognitive deficits is not addressed in this study, it should be noted that mir363-3p repressed caspase-3, which likely contributes to neuroprotection in the immediate aftermath of a stroke. We hypothesize that this early preservation of neurons may reduce secondary neuronal degeneration resulting from stroke, as seen in the white matter tracts. Some support for this hypothesis comes from our previous study, where mir363-3p treatment in the acute phase prevented the loss of mesostriatal projections in the ischemic hemisphere (Panta et al., 2019).

A positive diagnosis for a DSM V Neurocognitive disorder (Hugo and Ganguli, 2014) includes at least one of the following: complex attention, executive functioning, memory, language, perceptual-motor/visuospatial function, and social cognition. Changes in personality and social behavior can also occur with cognitive impairment (Viskontas and Miller, 2007; Rabinovici and Miller, 2010; Dillon et al., 2013). In the present study, non-spatial and visuospatial functions were modeled by the NORT and Barnes Maze test, respectively. In a previous study, we reported that NORT was not affected at 3 months after stroke, indicating that this non-spatial memory function possibly deteriorates between 3–6 months Remarkably, recognition of the novel object was retained in the mir363-3p treated groups. This pattern of stroke-induced cognitive impairment and protection by mir363-3p treatment was also seen in the Barnes maze analysis. Recognizing that motor impairment may confound some of the results on tests of neurocognitive assessment, our tests show that velocity in the Barnes maze was similar for stroke groups. Speed differences in Barnes are likely due to sensory rather than motor deficits. Impairment on the adhesive removal test does not persist after 30 days post-stroke (Panta et al., 2019), indicating that general sensory-motor function returns to normal. Furthermore, the gait analysis, using the Digigait apparatus, showed that all groups had similar gait and did not have any ambulatory difficulties pre and post-stroke, with the single exception of paw placement angle.

Despite the absence of any major motor deficits at 18 months of age (6 months post-stroke), gait analysis yielded an unexpected difference in the paw angle. As compared to sham, there was a bilateral narrowing of forelimb paw angle in the MCAo + scrambled group and a unilateral increase in paw angle on the contralesional hind limb. This was not seen in the group that received mir363-3p. Paw angle changes have been attributed to ataxia and demyelinating diseases (Powell et al., 1999). In the spontaneous mutation trembler J mouse, which is a model for early-onset demyelinating neuropathy, gait videography shows decreased forelimb paw angle and increased hindlimb paw angle (Falk et al., 2018). This is consistent with the present data where changes in the paw angle of the forelimb and hindlimb in the scrambled treated stroke group were also associated with a reduced volume of myelinated tracts in the ischemic hemisphere. White matter pathways are critical for cognitive functions (Fields, 2008; Chevalier et al., 2015; Vanes et al., 2019). Studies have suggested myelin breakdown occurs as a consequence of stroke and due to chronological aging (Guttmann et al., 1998; Peters et al., 2000; Bartzokis et al., 2001). Oligodendrocytes, in particular, are highly vulnerable to ischemia, resulting in demyelination after stroke (Back et al., 2002; Karadottir et al., 2005). In the present study, it is not possible to discriminate whether the reduced myelin volume is due to a loss of myelination or the loss of myelinated axons resulting from stroke-induced cell death. Given the evidence that mir363-3p reduced infarct volume and caspase activation in the acute stage of stroke (Selvamani and Sohrabji, 2017), it raises the possibility that preservation of myelin tracts may be due to limiting neuronal injury.

While this study shows that cognitive impairment occurs after stroke and can be abrogated by mir363-3p treatment, several issues remain unanswered. A critical limitation about clinical translation is whether mir363-3p can be effective as a primary treatment for cognitive impairment, or whether it has any off-target effects on the brain. To address this issue, a delayed administration of mir363-3p (weeks or months after stroke) or administration only after the animals start to show cognitive deficits would be needed. Additionally, multiple doses of mir363-3p may also be necessary. Furthermore, while mir363-3p is neuroprotective in the current stroke model, which mimics a stroke from a gradual thrombus formation from an atherosclerotic plaque, it is not known if it would have similar actions in stroke models where there is an abrupt occlusion (mimicking embolic stroke). Finally, our previous work showed that mir363-3p reduced infarction in a sex-specific manner by regulating neuronal caspase-3 activities only in females. As more is known about gene targets of mir363-3p, it would be worth investigating if a different dose or a cocktail of miRs would improve post-stroke cognitive function in males as well.

In conclusion, effective therapy for cognitive decline after a stroke remains an unmet medical need. These data suggest that early intervention after ischemia may prevent or alleviate stroke-induced cognitive impairment. It may be cautiously inferred that mir363-3p treatment in the acute phase, may have preserved or influenced cognitive resilience, as treated rats are similar to sham. It is unclear if treatment influenced spatial learning and memory (hippocampal-dependent) or perhaps attention and motivation (cortical function), and further studies are needed to determine the precise locus of mir363-3p action. A significant future direction for this work would be to determine if delayed mir363-3p treatment, given days or months after stroke, would have the same ability to rescue stroke-induced cognitive impairment.

## Data Availability Statement

The raw data supporting the conclusions of this article will be made available by the authors, without undue reservation.

## Ethics Statement

The animal study was reviewed and approved by Texas A&M University Institutional Animal Care and Use Committee.

## Author Contributions

AP: designed and performed the experiments; analyzed data and prepared figures, and wrote the manuscript. KM: supervised Barnes maze test, assisted with data interpretation, prepared figures, and contributed to manuscript preparation. MN: assisted with experiments and performed behavioral tests. KKM: performed gait analysis, prepared figures, and contributed to manuscript preparation. DS: performed histological analysis, prepared figures, and contributed to manuscript preparation. FS: conceptualized the study, analyzed data, and interpreted and wrote the manuscript. All authors contributed to the article and approved the submitted version.

## Conflict of Interest

The authors declare that the research was conducted in the absence of any commercial or financial relationships that could be construed as a potential conflict of interest.
