# Supplementary figures and images for: Mir363-3p Treatment Attenuates Long-Term Cognitive Deficits Precipitated by an Ischemic Stroke in Middle-Aged Female Rats
Source: Front Aging Neurosci. 2020 Sep 29;12:586362. doi: 10.3389/fnagi.2020.586362 (PMC7550720; doi:10.3389/fnagi.2020.586362)

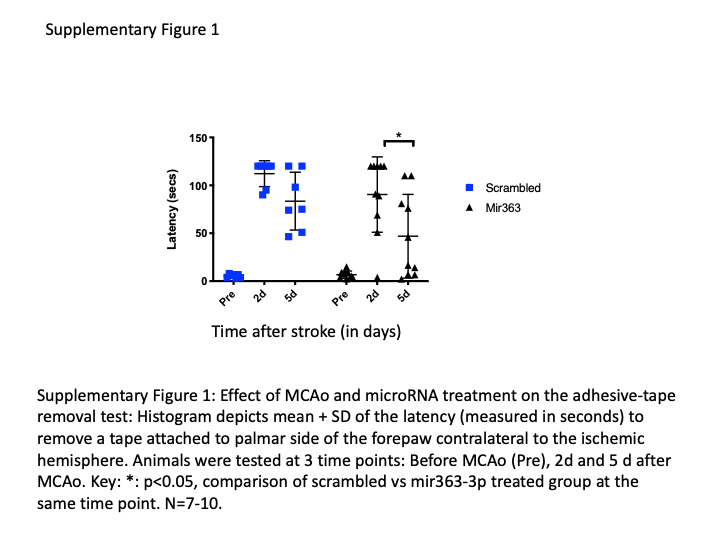

Supplement: Supplementary file 1 [file Image_1.TIFF]

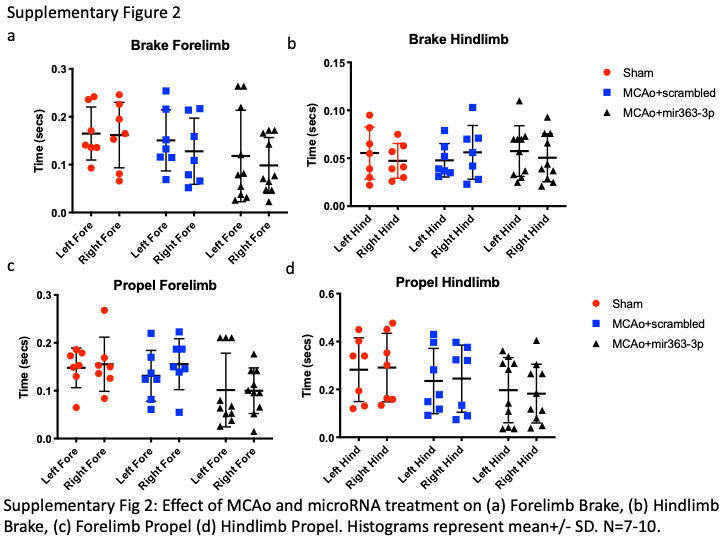

Supplement: Supplementary file 2 [file Image_2.TIFF]

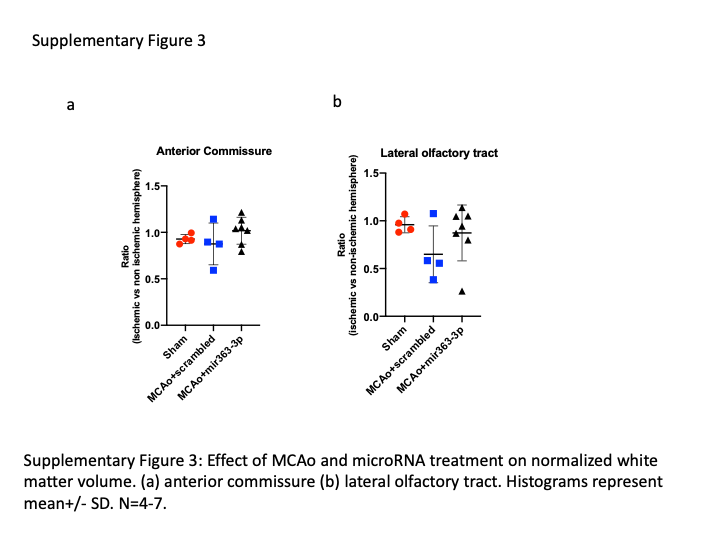

Supplement: Supplementary file 3 [file Image_3.TIFF]
